# Protocol for the Management of Metabolic and Cardiovascular Risk in Psychiatric Inpatients

**DOI:** 10.1192/j.eurpsy.2025.456

**Published:** 2025-08-26

**Authors:** J. Barreira, C. Rodrigues, D. Barbosa, S. Macedo

**Affiliations:** 1 Psychiatry and Mental Health; 2 Endocrinology and Nutrition; 3 Internal Medicine, Unidade Local de Saúde do Tâmega e Sousa, Porto, Portugal

## Abstract

**Introduction:**

Psychiatric patients, especially those with psychotic disorders, face an increased metabolic and cardiovascular risk, which ultimately leads to higher mortality from cardiovascular disease and reduced life expectancy. This is due to a multitude of risk factors, including those related to the course of the mental illness, lifestyle, socioeconomic and cultural circumstances, and the use of certain medications, such as atypical antipsychotics. This underscores the need for structured interventions during hospitalization to identify and manage these risks. Non-pharmacological interventions, such as physical activity and dietary education, have shown to be beneficial in managing weight and improving cardiovascular health. Pharmacological treatments, particularly the use of metformin and aripiprazol, have demonstrated efficacy in reducing metabolic disturbances such as weight gain, dyslipidemia, and hyperglycemia. The implementation of structured protocols to mitigate metabolic risk in psychiatric inpatients is recommended.

**Objectives:**

The main goal of the protocol is to guide the assessment, diagnosis, and therapeutic management of metabolic and cardiovascular risk factors in psychiatric inpatients. It also aims to guide the follow-up of these patients after discharge and coordination with other medical specialties.

**Methods:**

The protocol was developed in an interdisciplinary manner, involving the specialties of Psychiatry, Endocrinology, and Internal Medicine, and is based on recent guidelines and recommendations from other countries on this matter. The protocol was also adapted to the current conditions of the psychiatric inpatient setting.

**Results:**

The protocol involves a thorough clinical evaluation, including medical history, physical examination, and extensive laboratory analysis to identify comorbidities. Cardiovascular risk is calculated using the SCORE2, PRIMROSE and QRISK3 models. Non-pharmacological interventions include physical activity programs and nutritional counseling, while pharmacological interventions involve adjusting antipsychotic medications, selecting antipsychotics with more favourable metabolic profiles, and the adjuvant use of medications like metformin, aripiprazol, and topiramate. These evaluations are conducted at regular intervals, including post-discharge, to assess the efficacy of these interventions.

**Conclusions:**

Psychiatric inpatients, especially those on atypical antipsychotics, face significant metabolic and cardiovascular risks. A comprehensive assessment of risk factors and early intervention during hospitalization, combining lifestyle modifications and pharmacological treatments, can significantly reduce these risks and improve patient outcomes. Continued monitoring and coordination with outpatient services post-discharge are essential for sustained management of these health risks.

**Disclosure of Interest:**

None Declared

